# Osteoarthritis: The Most Common Joint Disease and Outcome of Sports Injury

**DOI:** 10.3390/jcm12155103

**Published:** 2023-08-03

**Authors:** Bowen Chen, Wei Huang, Junyi Liao

**Affiliations:** 1 Department of Orthopedic Surgery, The First Affiliated Hospital of Chongqing Medical University, Chongqing 400016, China; 2 Orthopedic Research Laboratory, Chongqing Medical University, Chongqing 400016, China

Osteoarthritis (OA) is the most common joint disease and affects an estimated 240 million people worldwide [1], and with the rapid aging of populations and growing challenge of obesity, the number of OA patients will continuously increase [2,3,4]. OA is characterized by articular cartilage wear and tear disorder and results in disability, loss of function, decreased quality of life (QoL), and economic burden. Except the aging and degeneration of the cartilage, sports-injury-caused instability of the joint would accelerate the progression of post-traumatic OA and OA of obesity [4]. Pain-modifying is the main management for early or middle stage OA, and joint replacement for end stage OA [5,6]. However, from early stage to end stage OA, most patients suffered pain and/or disability for more than ten years [5,6]. Therefore, clarifying the molecular mechanisms of OA and finding disease-modifying therapeutic strategies for osteoarthritis is urgent and essential for OA treatment.

As whole joint disease, pathological changes of OA are involved in cartilage (extracellular matrix, ECM, and chondrocytes), subchondral bone, and synovium.

First, as an aneural, avascular, and lymphatic structure, cartilage is composed of 65–80% water, 20–40% extracellular matrix (ECM), and 1–5% chondrocytes [7,8]. Cartilage degeneration is involved in chondrocytes apoptosis, aging, synthesis, and catabolism imbalance of ECM. In addition, joint appendicular structure injury was found to induce tear of cartilage, such as meniscus tear, abnormal anatomic-structure-induced repeated injury of the joint would accelerate the progress of OA [9,10,11,12]. 

Secondly, subchondral bone is the bedrock of joint cartilage and its remodeling plays a key role within pathogenesis and the progression of OA. As the junction of soft tissues and hard tissues, subchondral bone was identified as contributing to subchondral sclerosis, osteophytes formation and subchondral cysts formation [6,7,8,13]. Zhen et al. identified that the activation of TGFβ signaling in subchondral bone initiated the pathological changes of OA [14]. Furthermore, the activation of TGFβ signaling was proved to be associated with subchondral bone angiogenesis and H type vessel formation [14,15,16]. These results indicate TGFβ signaling may be the potential therapy targets of OA [14,15,16].

Third, a synovial hyperplasia-mediated chronic inflammation microenvironment was recently identified to play an essential role in OA initiation and progression [17,18,19]. It was found that synovial inflammation predates structural changes of the joints and positively correlated with the severity of OA [17,18,19]. Meanwhile, synovial inflammation affects degeneration of ECM, subchondral remodeling, and osteophyte formation [8,20]. A recent study identified the essential role of synovial fibroblasts in joint health and arthritis pathology, and it indicated that synovial fibroblast is a potential target for knee injury and degeneration treatment [21]. 

Collectively, cartilage degeneration, subchondral remodeling, and synovial inflammation are core pathological changes in OA. Although sports-related injuries may be the initial factor of OA, the “crosstalk” among the three pathological changes or tissue types is not clear, and the classification of OA is far from consistent. Therefore, further detailed studies clarifying the mechanism of OA development and progression will help us to explore potential targets for OA treatment.

We invite researchers to submit novel research articles, state-of-the-art reviews, and communications related to the fields of joint disease and sport medicine to this Special Issue. Such papers can focus on clarifying mechanisms of OA: sports injury management rehabilitation, physical therapy role in treating sports injuries, developments in sports injury epidemiology, sport activity levels, and revised joints surgery; association between sports injury and OA: diagnosis and treatment of joint disease and sports medicine; recent advancements in joint disease treatment, such as artificial joint replacement, joint arthroplasty; and biological materials. Additionally, sports medicine deals with the diagnosis, management, and treatment of injuries related to exercise or recreational activity. We look forward to receiving your contributions and to future collaborations.
